# Supplementary material for: Pyrethroid-piperonyl butoxide (PBO) nets reduce the efficacy of indoor residual spraying with pirimiphos-methyl against pyrethroid-resistant malaria vectors
Source: Sci Rep. 2022 Apr 27;12:6857. doi: 10.1038/s41598-022-10953-y (PMC9046380; doi:10.1038/s41598-022-10953-y)
Supplement: Supplementary file 1 — Supplementary Table S1. [file 41598_2022_10953_MOESM1_ESM.docx]

**Can pyrethroid-piperonyl butoxide (PBO) nets reduce the efficacy of indoor residual spraying with pirimiphos-methyl against pyrethroid-resistant malaria vectors?**

**Authors:** Thomas Syme^1,2,3^, Martial Gbegbo^2,3^, Dorothy Obuobi^1,2,3^, Augustin Fongnikin^2,3^, Abel Agbevo^2,3^, Damien Todjinou^2,3^, Corine Ngufor^1,2,3*^

**Supplementary information**

**Table S1: Detailed results from tunnel tests with pyrethroid-PBO and pyrethroid-only nets**

| **Strain** | **Susceptible *An. gambiae* Kisumu** | | | | | | **Pyrethroid-resistant *An gambiae* s.l. Covè** | | | | | |
| --- | --- | --- | --- | --- | --- | --- | --- | --- | --- | --- | --- | --- |
| **Net type** | **Untreated net** | **Olyset Net** | **PermaNet 2.0** | **Olyset Plus** | **PermaNet 3.0 (sides)** | **PermaNet 3.0 (roof)** | **Untreated net** | **Olyset Net** | **PermaNet 2.0** | **Olyset Plus** | **PermaNet 3.0 (sides)** | **PermaNet 3.0 (roof)** |
| **N exposed** | 307 | 191 | 218 | 191 | 196 | 229 | 208 | 300 | 216 | 303 | 184 | 200 |
| **N pass** | 236 | 65 | 44 | 11 | 58 | 109 | 107 | 69 | 59 | 53 | 103 | 108 |
| **% passage** | 76.9 | 34.0 | 20.2 | 5.8 | 29.6 | 47.6 | 51.4 | 23.0 | 27.3 | 17.5 | 56.0 | 54.0 |
| **95% CIs range** | 72.2-81.6 | 27.3-40.8 | 14.9-25.5 | 2.5-9.1 | 23.2-36 | 41.1-54.1 | 44.6-58.2 | 18.2-27.8 | 21.4-33.3 | 13.2-21.8 | 48.8-63.2 | 47.1-60.9 |
| **N blood fed** | 246.0 | 105.0 | 0.0 | 0.0 | 2.0 | 7.0 | 191.0 | 22.0 | 26.0 | 6.0 | 73.0 | 68.0 |
| **% blood fed** | 80.1 | 55.0 | 0.0 | 0.0 | 1.0 | 3.1 | 91.8 | 7.3 | 12.0 | 2.0 | 39.7 | 34.0 |
| **95% CIs range** | 75.7-84.6 | 47.9-62 | 0.0-5 | 0.0-5 | 0-2.4 | 0.8-5.3 | 88.1-95.6 | 4.4-10.3 | 7.7-16.4 | 0.4-3.6 | 32.6-46.7 | 27.4-40.6 |
| **% blood-feeding inhibition** | - | 31.4 | 100.0 | 100.0 | 98.7 | 96.2 | - | 92.0 | 86.9 | 97.8 | 56.8 | 63.0 |
| **N dead 24 h** | 34.0 | 187.0 | 218.0 | 190.0 | 196.0 | 227.0 | 5.0 | 125.0 | 58.0 | 272.0 | 62.0 | 136.0 |
| **% dead 24 h** | 11.1 | 97.9 | 100.0 | 99.5 | 100.0 | 99.1 | 2.4 | 41.7 | 26.9 | 89.8 | 33.7 | 68.0 |
| **95% CIs range** | 7.6-14.6 | 95.9-99.9 | 95-100.0 | 98.5-100.0 | 95-100.0 | 97.9-100.0 | 0.3-4.5 | 36.1-47.3 | 20.9-32.8 | 86.4-93.2 | 26.8-40.5 | 61.5-74.5 |
